# Servant leadership: an endangered species?

**DOI:** 10.1186/s13037-022-00321-0

**Published:** 2022-02-14

**Authors:** Philip F. Stahel, Niaz Ahankoob, Christine Nguyen

**Affiliations:** 1 College of Osteopathic Medicine, Rocky Vista University, 8401 S. Chambers Rd., Parker, CO 80134 USA; 2 The Medical Center of Aurora, 1501 S. Potomac St., Aurora, CO 80012 USA; 3 Arkansas College of Osteopathic Medicine, 7000 Chad Colley Blvd., Fort Smith, AR 72916 USA

**Keywords:** Leadership, Servitude, Graduate medical education, Patient safety

*“Power tends to corrupt, and absolute power corrupts absolutely.”* (John Emerich Edward Dalberg-Acton; 1834–1902). This historic quote by Lord Acton remains as applicable in the twenty-first century as in his original writing on moral authority in 1887 [1]. Individuals promoted into a leadership position may either (a) use their power as a *personal responsibility* to serve and influence others (“servant/transformative leaders”); (b) abuse their ability to control valued resources as a *personal freedom* to serve themselves (“autocratic/transactional leaders”); or (c) cling to their leadership position by avoiding controversy and decision-making in order to minimize the risk of making a wrong or unpopular decision (“indifferent leaders”). Arguably, there is also a hybrid combination of autocratic and indifferent leadership styles.

Servant leadership was first popularized by Robert Greenleaf in the 1970s [2] and has been traditionally defined as a leader with high moral authority who leads by conscience, not by ego [3]. At the core of this philosophy, servant leaders must be willing to sacrifice a position of power by serving instead of ruling others [4]. Their virtues are characterized by humility, integrity, accountability, gratitude, empathy, and a desire to serve a higher purpose beyond their own selves [3]. One of the core characteristics of servant leadership is “active” listening [5]. Active listening skills are an extension of empathy and compassion, whereby the “empathetic listener” fully concentrates, attempts to understand, remembers what is communicated, and responds with “readbacks” by paraphrasing what the other person said [6]. Active listening involves both verbal and nonverbal techniques to demonstrate that the listener is truly paying attention [5]. In contrast, “passive” listening does not require any more effort than simply hearing what is being said [5]. Passive listening is reflective of an indifferent or autocratic leader who has no intention of understanding the other person. Empathy, defined as the ability to understand and share the feelings of another person (regardless whether we agree with that person’s perspective) represents the most important virtue of a servant leader [5, 7]. Neither empathy nor servitude can be playacted, and subordinates will invariably notice the difference between genuine empathetic servitude versus autocratic or indifferent leadership. The latter two leadership styles serve the sole purpose for individuals to retain and expand their position of power.

The key driver for an autocratic leader *(“What’s in it for me?”)* or indifferent leader *(“How can I avoid controversy?”)* is contrary to the desire to serve others under the paradigm of servant leadership *(“What is wanted of me?”)*. Interestingly, weak leaders who feel insecure in their position and doubt their own competence tend to embrace a more autocratic leadership style in order to control the potential threat to their power by others. These leaders will request unconditional loyalty from their subordinates in absence of reciprocity. Indifferent leaders are typically elevated or “grandfathered” into a leadership position through personal relationships, in absence of natural authority, merit, or a commitment to servitude. Such leaders will cling to their leadership position by avoiding controversy and proactive decision-making (“flying under the radar” paradigm) in order not to take the risk of making a wrong or unpopular decision. In contrast, servant leaders tend to earn genuine loyalty from their followers who understand that such leaders invariably act in their best interest.

In the business world, the autocratic model reflects a “top-down” hierarchy whereby a leader’s power is controlled through a paycheck. Under this paradigm, team members obey their leaders by performing “duties as assigned” in order to retain their job security [5]. In contrast, the principle of servant leadership is based on moral authority and governed by the individual leader’s personal conscience, sacrifice, and commitment to doing what is right for their community and constituents. Under this paradigm, the team members follow their servant leaders by feeling empowered and accountable for their own actions with the intent to contribute and to improve the organization.

In politics, autocratic leadership is represented by dictatorship. This model works most efficiently in times of crisis and military conflict by defining a “chain of command” with unequivocal decision-making authority. While beneficial for the sake of efficiency, the autocratic leadership model largely promotes mediocrity at the price of individual accountability. Intriguingly, such a leadership style can also impose a double-edge sword if subordinates follow a strict chain of command. This notion has been historically corroborated by the French military defeat at Waterloo on June 18, 1815. Napoleon Bonaparte had ordered his general Emmanuel de Grouchy to pursue the retreated Prussians. While witnessing the French army under massive attack by two of the armies of the Seventh Coalition, Grouchy continued to blindly follow the Emperor’s order instead of rushing to the French army’s salvation [8]. Napoleon and his army were defeated and suffered tens of thousands of causalities and losses [8]. The outcome of the landmark battle could have been turned around if Napoleon’s general had felt empowered to make a proactive “ad hoc” decision against the Emperor’s outdated orders. This historic example emphasizes the adverse impact of autocratic leadership (Napoleon) when weak subordinate leaders (Grouchy) do not feel empowered by decision-making authority.

Dr. Martin Luther King Jr. suitably stated that *“The ultimate measure of a man is not where he stands in moments of comfort or convenience, but where he stands at times of challenge and controversy”* [9]. This quote reflects on the observation that, unlike autocratic and indifferent leaders, servant leaders will step outside of their own comfort zone by aspiring to be “comfortable with being uncomfortable” for the sake of their constituents or organizations.

Outside of business and politics, the stratification of leadership styles (Table 1) can also be extrapolated to the field of medicine [10–12]. In modern healthcare, autocratic or indifferent leadership can negatively impact care delivery and assurance of quality and patient safety. Beyond the outdated entity of the “disruptive surgeon” who rules the operating room staff by fear and intimidation, patient safety can be more dramatically impacted on a larger scale by subtle nuances in authoritarian leadership at the level of physician leaders, department chairs, or hospital executives. Once autocratic leadership is introduced in the healthcare setting, staff and colleagues will feel deprived of an opportunity for providing meaningful work, pursuing personal growth, and speaking up on behalf of patient safety. The ensuing “brain drain” of competent and engaged colleagues who seek new opportunities elsewhere further exacerbates the downward spiral towards an evolution of “silo mentality” and erosion of trust, engagement, and individual accountability. A classic quote from leadership coaching states that *“People leave their bosses, not their companies.”*[13]

**Table 1:** Characteristics of prevalent leadership models

	**Servant/** **transformative leader**	**Autocratic/ transactional leader**	**Indifferent leader**
Leadership paradigm	“What’s needed of me?” (Empathy, servitude)	“What’s in it for me?” (Entitlement, abuse of power)	“How can I avoid controversy?” (Indifference)
Subordinates’ perspective	“How can I support this leader?” (Loyalty)	“How can I avoid this leader?” (Fear)	“How can I take advantage of this leader?” (Opportunism)
Leadership style	Credible role model	Dictatorship	Invisible
Decision-making style	Fosters argument and debate; takes calculated risks; involves others in decision-making	“Top-down” chain of command; imposes decisions on others	Risk-averse; avoids decision-making
Listening skills	Active empathetic listener	Passive listener	Passive/indifferent listener
Culture	“Just culture”	“Blame and shame”	“Flying under the radar”
How this leader makes you feel	Encouraged	Incompetent	Irrelevant

Beyond the effect on the delivery of clinical care, the quality of physician leadership also has a significant impact on graduate medical education and the training of the next generation of physicians [14]. Medical students undergo rigorous training and endure a range of triumphs and failures. In addition to navigating through the ups and downs of the curriculum and rotations, medical students are trying to figure out how they are going to leave their mark on this world and on patient’s lives. Medical students typically navigate the hardship and adversity of their training with the support of peers, and possibly with a student mentor from a class above, but rarely with the support of a designated physician mentor. After consulting with many student peers from medical schools across the United States, there is a general consensus that having a dedicated physician mentor is rare (Ahankoob and Nguyen, unpublished observations). Even the physicians who dedicate time to preceptorship and teaching only do so for the short time the student is rotating, anywhere from two to six weeks. Once the rotation is complete, most preceptors and students no longer maintain a mentorship relationship. The root cause of this apparent shortage of physician mentors for medical students is likely based on mutated values for practicing physicians in the modern age. Historically, the education of the next generation of physicians represented a non-negotiable noble duty that was part of the calling of the “servant heart” of a clinician and academician [4, 15, 16]. However, the paradigm of the “modern-day healer” has been transformed largely to shift work whereby non-compensated academic efforts may no longer be considered valuable as part of a meaningful “work-life-balance”.

Physicians are under increasing pressure to provide cost-efficient care while improving patient experience and outcomes, decreasing hospital length of stay, adhering to regulatory-compliance mandated standards of care, and providing pristine electronic health record documentation under the growing peril of medicolegal repercussions [17]. Under this evolving paradigm, physicians may no longer see a value proposition by dedicating valuable time towards leading and educating medical students, unless the teaching effort is financially incentivized as part of a preceptorship contract.

The rapidly expanding demands of healthcare also increase the amount of mental pressure and stress on medical students. The combination of working exceedingly hard, yet falling short in standardized tests, scribing medical notes instead of performing physical exams, receiving non-constructive criticism from preceptors, and being confronted with the looming risk of not matching a “dream specialty” contributes to the highly prevalent “impostor syndrome” among medical students, ultimately leading to burnout and depression, and even regret for pursuing the calling to become a physician. As a coping mechanism, empathy and compassion, the main motivators of medical school applicants, have been documented to erode in the 3^rd^ year of medical school [18]. This apparent paradox arrives at a crucial time in a medical student’s academic curriculum when the training shifts from cadaver dissection and classroom lectures to direct patient care and clinical work. While individuals who embark on the journey to become physicians likely do so with enthusiasm, idealism, and a genuine intention to become servants for vulnerable people in need, these high ideals appear to dramatically transform into cynicism and disillusionment towards the end of medical school [7]. Such experiences may be reflective of the notion that servant leadership in graduate medical education factually represents an “endangered species.”

The authors have personally witnessed several occurrences when medical students were deprived of an opportunity for a desired clerkship because the respective physician preceptor refused to mentor students in absence of a paid teaching contract or find no value in academic education because this is not what they “signed up for” as part of their clinical practice (Stahel, Ahankoob, Nguyen; unpublished observations). This assumption is substantiated by the following recent experience from a medical student: *“I have come to realize that dedicated and motivated physician mentors are hard to find, particularly in absence of monetary compensation. Similar to children being raised by their parents, medical students will always remember how their mentors made them feel during a time when they were most vulnerable and doubting themselves on the path to becoming servant leaders and healers. In return, today’s students will strive to return the duty by precepting and being a mentor to the next generation of medical students in the future.”*

Arguably, medical student burnout and impostor syndrome could be prevented with the guidance of genuine physician mentors who embrace the model of servant leadership by guiding, leading and teaching out of sense of duty in absence of other incentives [19–21]. The elusive servant leadership model may ultimately create a “mass effect” whereby young doctors would feel compelled to act as dedicated mentors and role models of servant leadership to help guide and provide a moral compass to the next generation of physicians, in perpetuity.

## Data Availability

Please contact the authors for data requests.
